# Data health risk assessment of nitrate contamination in groundwater of rural region in the Yerraguntla Mandal, South India

**DOI:** 10.1016/j.dib.2020.105374

**Published:** 2020-03-11

**Authors:** B. Suvarna, V. Sunitha, Y. Sudharshan Reddy, N. Ramakrishna Reddy

**Affiliations:** a Department of Geology, Yogi Vemana University, 516005, India; b Department of Geology, Loyola Degree College, 516390, India

**Keywords:** Nitrates, Health risk, Groundwater, Yerraguntla

## Abstract

The main objective of the present study was to determine the distribution levels of nitrate contamination in groundwater and its associated impact on human health risk in the Yerraguntla mandal, South India. For this 40 ground water samples were collected randomly during April 2018. Nitrate concentration in groundwater samples ranged from 2.50 to 760.12 mg/L, with a mean value of 86.13 mg/L. Most of the groundwater samples are exceeding the permissible limits of nitrate (45 mg/l). Hence health risk assessment of nitrate has been carried out. Hazard quotient (HQ) values for infants, children, male and female ranges from 0.05 to 14.25; 0.06 to 18.53; 0.04 to 12.18 and 0.05 to 14.62, respectively. The finding of data showed that HQ value was more than 1 in 42.5% of samples in groups of infants, children and female, and 35% of samples in group of male.

Specification table

**Table:** 

Subject area	Yerraguntla
More specific subject area	Nitrate in groundwater
Type of data	Tables and figures
How data was acquired	40 Groundwater Samples collected in different bore wells at Yerraguntla Mandal, Y.S.R district, A.P with help of location map.
	Groundwater samples analyzed using a UV–visible spectro-Photometer according to standard methods for examination of groundwater
Data format	Raw and analyzed
Parameters for data collection	Groundwater samples were tightly packed in 2 L
	Polyethylene plastic bottles, stored in Geo chemical lab at room
	Temperature and determined for nitrate concentration.
Description for data collection	Determine the concentration levels of nitrate
Data source Location	Yerraguntla Mandal, Y.S.R Kadapa District
Data accessibility	Data is included in this Article

**Value of the data**

• The data of present paper revealed that the nitrate concentration in 45% of groundwater samples exceeding the maximum permissible limits (45 mg/L) according to world health organization (WHO) guidelines.
• The findings of this study reveal that all the four groups i.e. infants, children, female and male are exposed to nitrate risk (HQ>1).
• High nitrate concentration in drinking water causes health risks on human body like methemoglobinemia in infants and stomach cancer in children.
• The findings of this study reveal the all the four groups i.e. infants, children, female and male are exposed to nitrate risk (HQ>1).
• Health risk assessment revealed that male, are less prone to risk than female. Hence proper precautionary measures have to be taken to control health risk in this area.

## 1 Data description

### 1.1 Study area

**Fig. 1:**
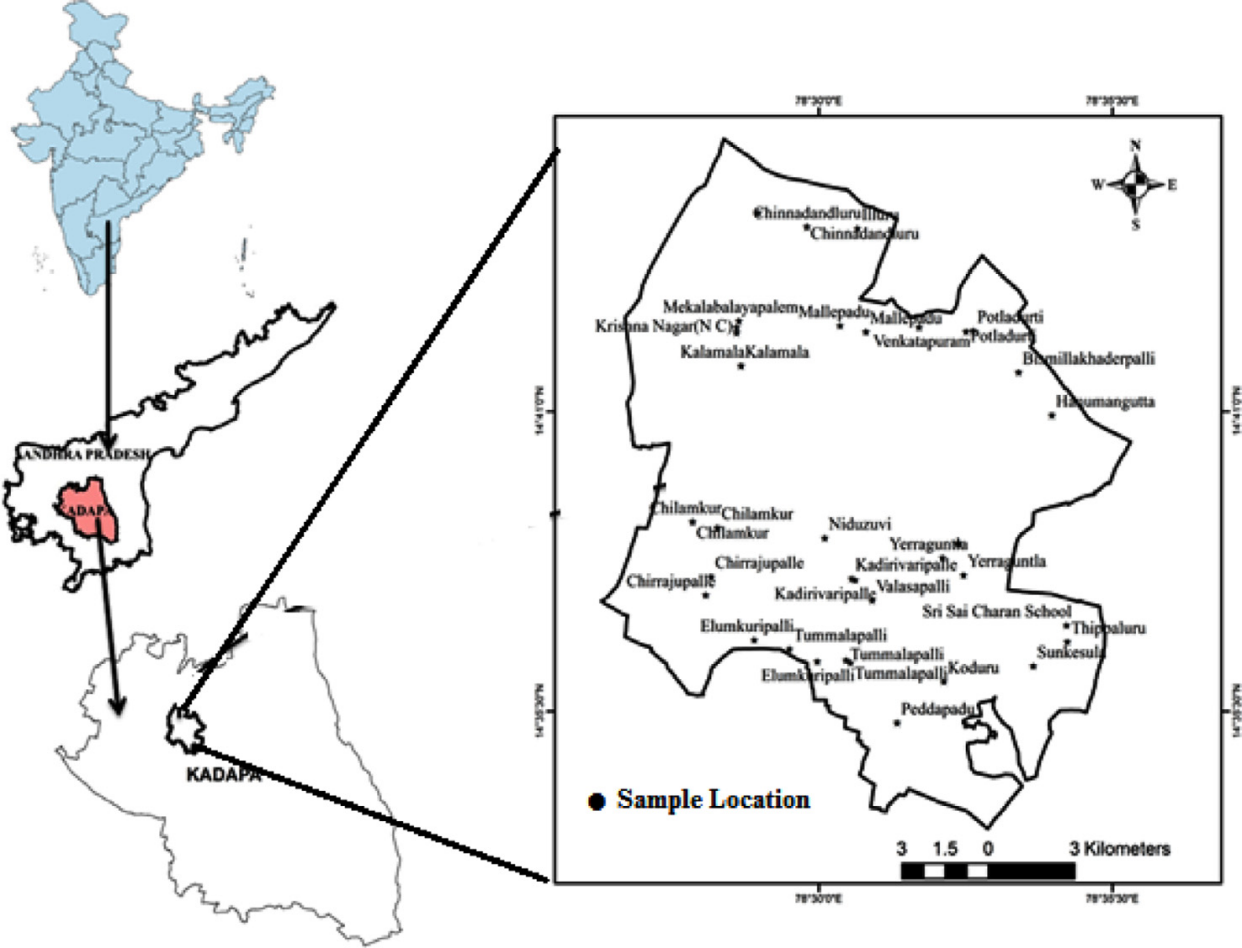
Location map of the study area (Yerraguntla).

Yerraguntla is a limestone area located within the Kadapa district of the Andhra Pradesh. The study area lies between Latitudes N 14°35ˊ 17.2ˊˊ–N 14°44ˊ 38.4ˊˊ and Longitudes E 78°27ˊ38.4ˊˊ–E 78°34ˊ39.3ˊˊ (Fig. 1). In this area mainly Arenaceous consisting of conglomerate quartzite, Quartzite with shale formation of dolomitic lime stones are present. Groundwater quality may vary depending upon variations in geological formations. The total annual rainfall in the study area is 730.7 mm [1,2].

### 1.2 Analytical data

**Fig. 2:**
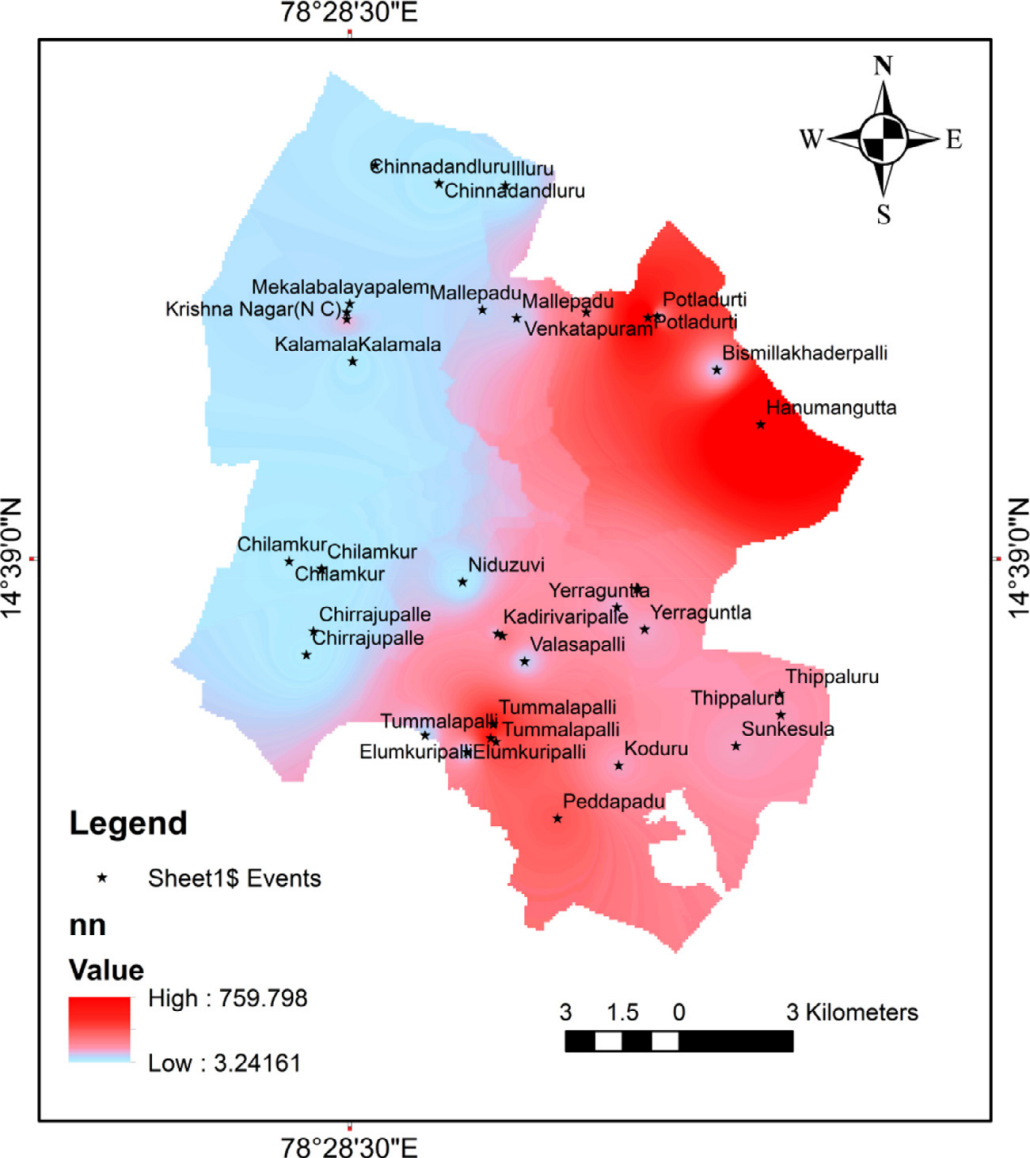
Spatial distribution map of nitrate concentration.

**Fig. 3:**
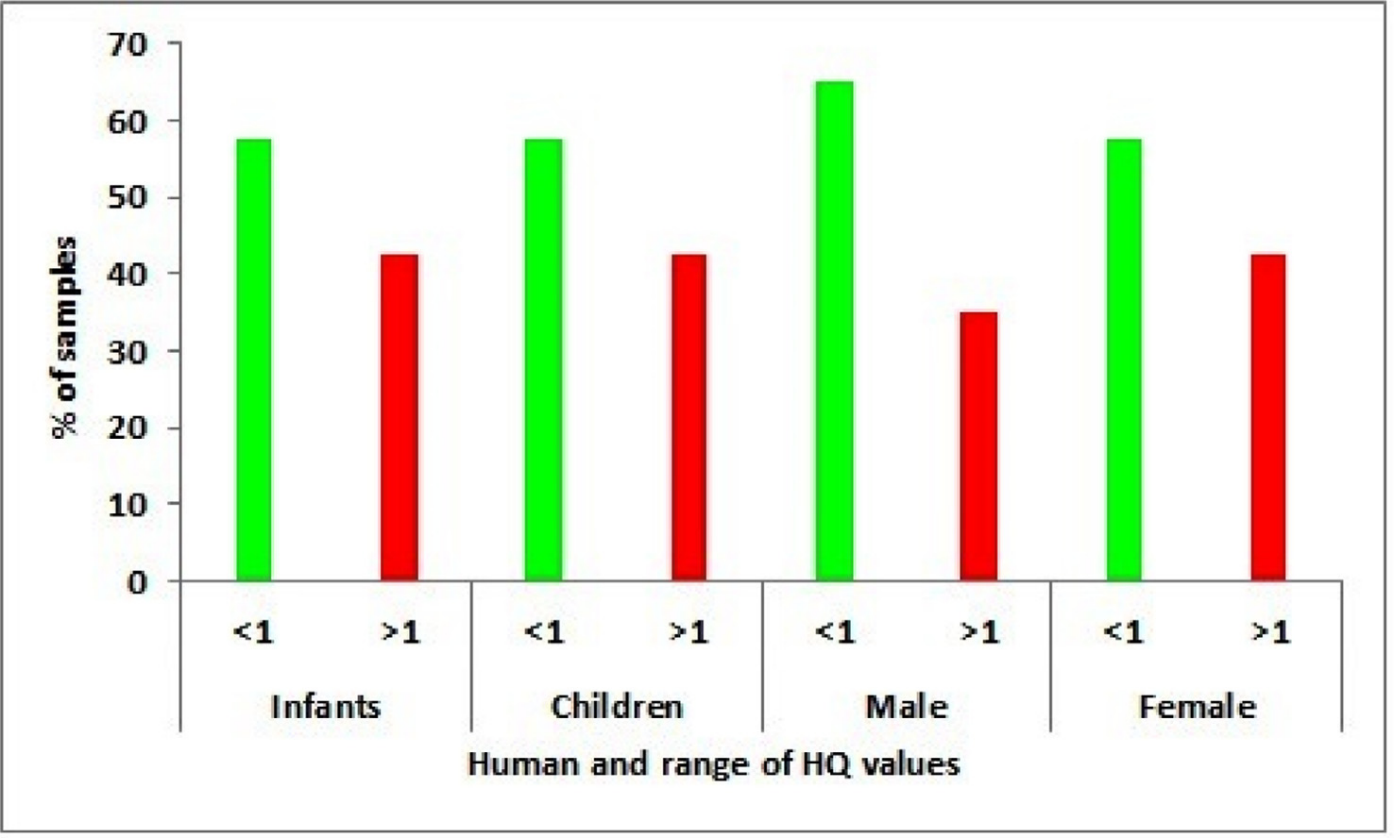
Frequency distribution of HQ nitrate concentration for four groups.

Spatial distribution map of the nitrate shown in Fig. 2; revealed that most of the higher nitrate concentration fall eastern part of the study area. The concentration of nitrate and HQ values for infants, children, female, male shown in Table 1. Table 2 show that the ranges of HQ for four groups. In the present study, 42.5% of the groundwater samples exceeding 1(HQ>1) is referred as adverse non-carcinogenic risk for human health, for infants, children, female and 35% of samplers exceeding 1(HQ>1) for male as shown in Fig. 3.

**Table 1:** Nitrate concentration and hazard quotient for the four groups of water consumers.

S. No	Name of the villages	Latitude	Longitude	Nitrate concentration (mg/L)	HQ Values			
					Infants	Children	Male	Female
1	Thippaluru	N 14°36ˊ 46.3ˊˊ	E 78°34ˊ39.3ˊˊ	69.2	1.30	1.69	1.11	1.33
2	Sri Sai Charan School	N 14°37ˊ 04.6ˊˊ	E78°34ˊ 38.4ˊˊ	72.2	1.35	1.76	1.16	1.39
3	Sunkesula	N 14° 36ˊ 19.7ˊˊ	E78°34ˊ 1.0ˊˊ	65.23	1.22	1.59	1.05	1.25
4	Koduru	N 14°36ˊ 2.7ˊˊ	E78°32ˊ20.6ˊˊ	65.34	1.23	1.59	1.05	1.26
5	Peddapadu	N 14°35ˊ17.2ˊˊ	E 78°31ˊ28.1ˊˊ	160.23	3.00	3.91	2.57	3.08
6	Tummalapalli	N 14°36ˊ23.3ˊˊ	E 78°30ˊ35.6ˊˊ	140.36	2.63	3.42	2.25	2.70
7	Tummalapalli	N 14°36ˊ26.2ˊˊ	E 78°30ˊ 30.9ˊˊ	290.24	5.44	7.07	4.65	5.58
8	Elumkuripalli	N 14°36ˊ38.3ˊˊ	E 78°30ˊ 32.9ˊˊ	250.24	4.69	6.10	4.01	4.81
9	Elumkuripalli	N 14°36ˊ 1.5ˊˊ	E 78°29ˊ 30.7ˊˊ	28	0.53	0.68	0.45	0.54
10	Tummalapalli	N 14°35ˊ51.7ˊˊ	E 78°29ˊ 5.1ˊˊ	26	0.49	0.63	0.42	0.50
11	Valasapalli	N 14°37ˊ 32.2ˊˊ	E 78°31ˊ 0ˊˊ	36	0.68	0.88	0.58	0.69
12	Kadirivaripalle	N 14°37ˊ 54.4ˊˊ	E 78°30ˊ 40.9ˊˊ	140.24	2.63	3.42	2.25	2.70
13	Kadirivaripalle	N 14°37ˊ 55.9ˊˊ	E 78°30ˊ 37.0ˊˊ	36	0.68	0.88	0.58	0.69
14	Chirrajupalle	N 14°37ˊ 37.9ˊˊ	E 78°27ˊ 53.2ˊˊ	3.42	0.06	0.08	0.05	0.07
15	Chirrajupalle	N 14°37ˊ 58.01ˊˊ	E 78°27ˊ59.2ˊˊ	9	0.17	0.22	0.14	0.17
16	Chilamkur	N 14°38ˊ 51.3ˊˊ	E 78°28ˊ 6.4ˊˊ	2.5	0.05	0.06	0.04	0.05
17	Chilamkur	N 14°38ˊ 58.3ˊˊ	E 78°27ˊ 38.4ˊˊ	6.3	0.12	0.15	0.10	0.12
18	Chilamkur	N 14°38ˊ 58.3ˊˊ	E 78°27ˊ 38.4ˊˊ	4.21	0.08	0.10	0.07	0.08
19	Niduzuvi	N 14°38ˊ 52.0ˊˊ	E 78°28ˊ 6.2ˊˊ	9.24	0.17	0.23	0.15	0.18
20	Chilamkur	N 14°38ˊ40.6ˊˊ	E 78°30ˊ 6.9ˊˊ	8.2	0.15	0.20	0.13	0.16
21	Kalamala	N 14°41ˊ 50.1ˊˊ	E 78°28ˊ 32.9ˊˊ	10.12	0.19	0.25	0.16	0.19
22	Kalamala	N 14°41ˊ 50.0ˊˊ	E 78°28ˊ 32.7ˊˊ	10.24	0.19	0.25	0.16	0.20
23	Krishna Nagar(N C)	N 14°42ˊ 32.3ˊˊ	E 78°28ˊ 27.7ˊˊ	11.2	0.21	0.27	0.18	0.22
24	Mekalabalayapalem	N 14°42ˊ 39.5ˊˊ	E 78°28ˊ 30.3ˊ	17.24	0.32	0.42	0.28	0.33
25	Chinnadandluru	N 14°44ˊ 38.4ˊˊ	E 78°28ˊ 52.2ˊˊ	15.24	0.29	0.37	0.24	0.29
26	Chinnadandluru	N 14°44ˊ 22.8ˊˊ	E 78°29ˊ 46.6ˊˊ	9.24	0.17	0.23	0.15	0.18
27	Chinnadandluru	N 14°44ˊ 22.8ˊˊ	E 78°29ˊ 46.9ˊˊ	10.25	0.19	0.25	0.16	0.20
28	Illuru	N 14^0^44ˊ 21.1ˊˊ	E 78°30ˊ 43.5ˊˊ	11.24	0.21	0.27	0.18	0.22
29	Mallepadu	N 14°42ˊ 34.3ˊˊ	E 78°30ˊ 23.9ˊˊ	32.12	0.60	0.78	0.51	0.62
30	Mallepadu	N 14°42ˊ 27.3ˊˊ	E 78°30ˊ 53.1ˊˊ	36.26	0.68	0.88	0.58	0.70
31	Venkatapuram	N 14°42ˊ 32.3ˊˊ	E 78°31ˊ 52.8ˊˊ	110.24	2.07	2.69	1.77	2.12
32	Potladurti	N 14°42ˊ 27.6ˊˊ	E 78°32ˊ 45.6ˊˊ	560.24	10.50	13.66	8.98	10.77
33	Potladurti	N 14°42ˊ 28.5ˊˊ	E 78°32ˊ 53.5ˊˊ	26	0.49	0.63	0.42	0.50
34	Bismillakhaderpalli	N 14°41ˊ 42.8ˊˊ	E 78°33ˊ 44.6ˊˊ	40	0.75	0.98	0.64	0.77
35	Hanumangutta	N 14°40ˊ 55.7ˊˊ	E 78°34ˊ 22.0ˊˊ	760.12	14.25	18.53	12.18	14.62
36	Yerraguntla	N 14°38ˊ 35.0ˊˊ	E 78°32ˊ 37.5ˊˊ	120.24	2.25	2.93	1.93	2.31
37	Yerraguntla	N 14°38ˊ 34.2ˊˊ	E 78°32ˊ 35.9ˊˊ	58	1.09	1.41	0.93	1.12
38	Yerraguntla	N 14°38ˊ 18.8ˊˊ	E 78°32ˊ 19.1ˊˊ	56	1.05	1.37	0.90	1.08
39	Yerraguntla	N 14°37ˊ 59.8ˊˊ	E 78°32ˊ 42.8ˊˊ	62	1.16	1.51	0.99	1.19
40	Thermal waste water	N 14°42ˊ 26.3ˊˊ	E 78°28ˊ 27.7ˊˊ	67	1.26	1.63	1.07	1.29

**Table 2:** Classification of HQ ranges for Infants, children, male, female.

Human	Range of HQ	Health risk	Sample numbers	No of samples	% of samples
Infants	<1	No risk	9 to 11, 13 to 30, 33, 34	23	57.5
	>1	High risk	1 to 8, 12, 31, 32, 35 to 40	17	42.5
Children	<1	No risk	9 to 11, 13 to 30, 33, 34	23	57.5
	>1	High risk	1 to 8, 12, 31, 32, 35 to 40	17	42.5
Male	<1	No risk	9 to 11, 13 to 30, 33, 34, 37, 38, 39	26	65
	>1	High risk	1 to 8, 12, 31, 32, 35, 36, 40	14	35
Female	<1	No risk	9 to 11, 13 to 30, 33, 34	23	57.5
	>1	High risk	1 to 8, 12, 31, 32, 35 to 40	17	42.5

## 2 Experimental design, materials, and methods

The Samples were collected from 40 points in the different villages of Yerraguntla Mandal (Fig. 1). The samples were collected systematically during the month of April from bore/ hand pumps of study area. The samples were collected in acid washed 1 litter polyethylene bottles to prevent unpredictable changes in characteristic as per standard procedures [3]. Each of the groundwater samples was analyzed for nitrate. The nitrate concentration was measured using Spectro- photometer accordance standard methods for examination of groundwater [4], [5], [6], [7].

### 2.1 Human health risk assessment

In recent decades, groundwater quality has continued to deteriorate due to the various pollution sources such as chemicals and fertilizers [8]. And due to this there is an increased awareness of health risk on humans. Health risk assessment plays a crucial role in this new era. Health assessment insures and evaluates adverse human health effect on infants, children, female and male [9,10]. This study signifies the health risk assessment associated with NO_3_^−^ in drinking water and around Yerraguntla Mandal. The chronic daily intake (CDI) and hazard quotient (HQ) were calculated by following formulas:

$$\text{CDI}=\frac{\text{CPW}\times \text{IR}\times \text{ED}\times \text{EF}}{\text{ABW}\times \text{AET}}$$

$$\text{HQ}=\frac{\text{CDI}}{\text{RfD}}$$

Where CDI is the chronic daily intake (mg/kg/day), CPW indicate nitrate contamination concentration in groundwater (mg/L), Ingestion rate (IR) 2 L/day for male and female, 0.78 L/day for children, 0.3 L/day infants [11]. ED indicates exposure duration Years 40, 40,12 &<1 for male, female, children, infants respectively [12]. EF indicates exposure frequency is assigned, 365days/year for male, female, children and Infants, ABW denotes average body weight of human body in kg 78 (male), 65 (female), 20 (Children), 10 (infants), AET denotes average exposure time Days, 14,600 for male and female, 4380 children and 365 infants. RfD indicates that reference of NO_3_^−^ (1.6 mg/kg/d)were obtained from the database of Integrated Risk Information System (IRIS), and USEPA (2012) [13]. Hazard quotient value exceeding 1 is referred as adverse non-carcinogenic risk for human health, while HQ values less than 1 indicates acceptable limit of non-carcinogenic risk.

## Conflict of Interest

We declare that we have no known competing financial interests or personal relationships that could have appeared to influence the work reported in this paper.

## Appendix Supplementary materials

Image, application 1
